# How Accurate Appraisal of Behavioral Costs and Benefits Guides Adaptive Pain Coping

**DOI:** 10.3389/fpsyt.2017.00103

**Published:** 2017-06-12

**Authors:** Wiebke Gandhi, India Morrison, Petra Schweinhardt

**Affiliations:** ^1^Faculty of Dentistry, McGill University, Montreal, QC, Canada; ^2^The Alan Edwards Center for Research on Pain, McGill University, Montreal, QC, Canada; ^3^School of Psychology and Clinical Language Sciences, Centre for Integrative Neuroscience and Neurodynamics, University of Reading, Reading, United Kingdom; ^4^Center for Affective and Social Neuroscience, Department of Clinical and Experimental Medicine, Linköping University, Linköping, Sweden; ^5^Faculty of Medicine, Department of Neurology and Neurosurgery, McGill University, Montreal, QC, Canada; ^6^Interdisciplinary Spinal Research Group, Balgrist University Hospital, Zurich, Switzerland

**Keywords:** behavioral control, active compensatory coping, adaptive pain coping, maladaptive pain coping, mesolimbic dopamine system

## Abstract

Coping with pain is a complex phenomenon encompassing a variety of behavioral responses and a large network of underlying neural circuits. Whether pain coping is adaptive or maladaptive depends on the type of pain (e.g., escapable or inescapable), personal factors (e.g., individual experiences with coping strategies in the past), and situational circumstances. Keeping these factors in mind, costs and benefits of different strategies have to be appraised and will guide behavioral decisions in the face of pain. In this review we present pain coping as an unconscious decision-making process during which accurately evaluated costs and benefits lead to adaptive pain coping behavior. We emphasize the importance of passive coping as an adaptive strategy when dealing with ongoing pain and thus go beyond the common view of passivity as a default state of helplessness. In combination with passive pain coping, we highlight the role of the reward system in reestablishing affective homeostasis and discuss existing evidence on a behavioral and neural level. We further present neural circuits involved in the decision-making process of pain coping when circumstances are ambiguous and, therefore, costs and benefits are difficult to anticipate. Finally, we address the wider implications of this topic by discussing its relevance for chronic pain patients.

## Introduction

Pain is per definition an unpleasant sensation and signals (potential) harm to the organism (1). It therefore requires attention and needs to be addressed by the individual. Evidence suggests that pain triggers behavioral coping responses—aiming to reduce unpleasantness and harm, and to reach the best hedonic state possible within the given situation. In this article we discuss a variety of behaviors that are frequently exhibited to cope with a painful event. We further attempt to disentangle how characteristics of the painful stimulus itself as well as situational and personal factors influence the appraisal of costs and benefits, which ultimately determines the choice of a particular coping strategy *via* a complex network of relevant neural circuits.

**Figure 1:**
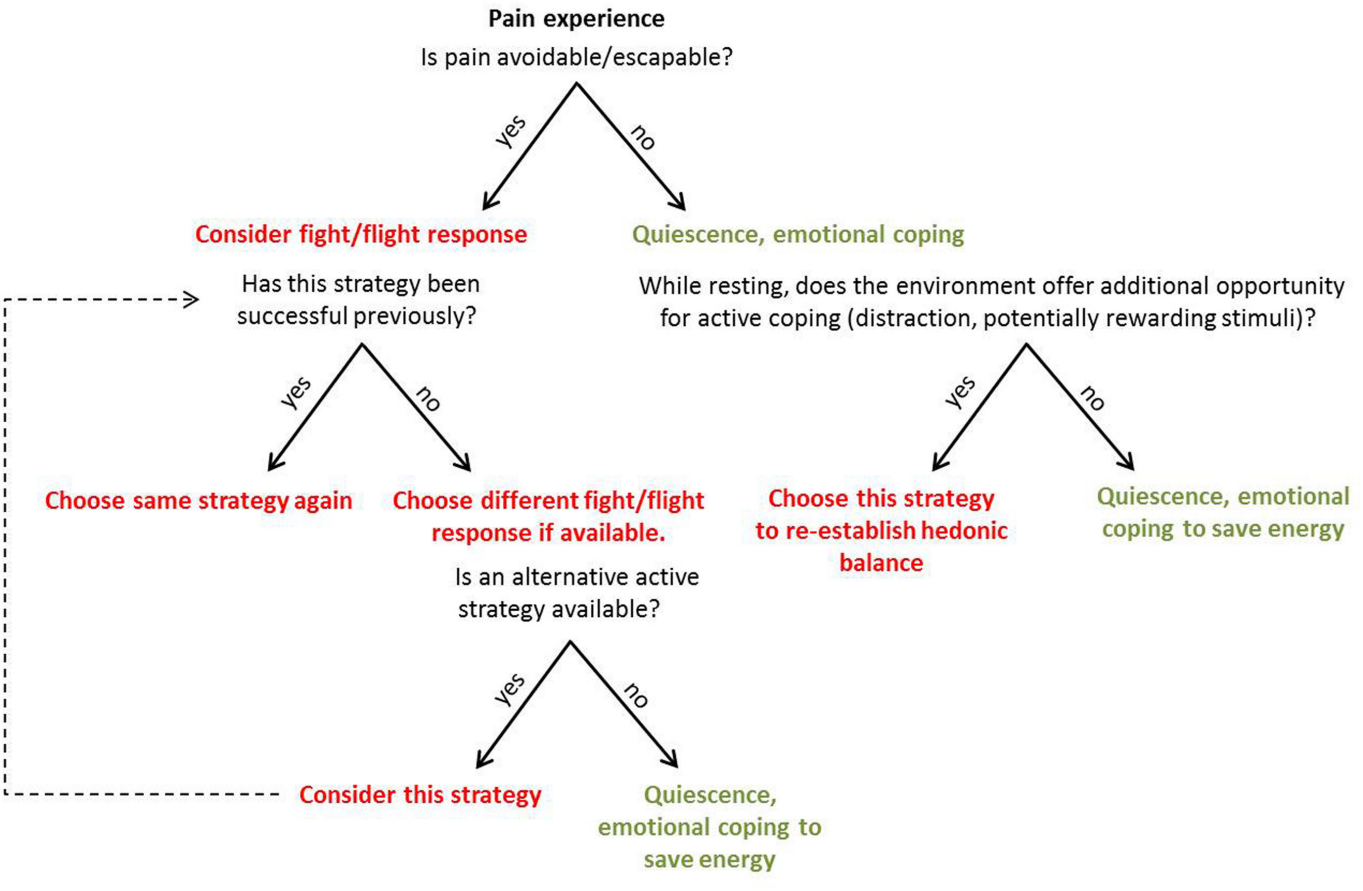
Representation of the suggested appraisal and unconscious decision-making process when an individual is faced with pain. The appraisal of costs and expected benefits is based on attributes of the pain itself, as well as personal and situational factors. Active coping strategies are represented in red, passive strategies in green.

Coping with pain arguably serves two objectives: to (i) eliminate or reduce pain and (ii) continue to pursue valued activities despite pain (2). During *active* coping, the organism focusses on external stimuli and addresses them directly (3). Major active coping strategies encompass behaviors such as escaping, avoiding, or attacking the source of pain (4), but possibly also actions aimed to offset the unpleasant component of pain *via* active pursuit of pleasurable stimuli or events in the environment. During *passive* coping, the organism shows decreased responsiveness to external stimuli and focusses on affect regulation (3). Major passive coping strategies are quiescence, pain acceptance, and forms of surrender (including relegating the locus of control to external forces). Active and passive coping strategies may prove beneficial or disadvantageous depending not only on characteristics of the pain itself (i.e., whether it is objectively escapable or not) but also on personal factors (individual learning history with similar situations) and situational aspects, such as opportunities provided by the environment to allow for certain strategies (Figure 1). The organism has to (unconsciously) appraise the behavioral costs and the anticipated benefits for the different available strategies to (unconsciously) choose the most economical one. An accurate appraisal of the cost/benefit ratio will guide adaptive pain coping behavior. For pain that is escapable, active coping strategies are likely to be adaptive, because the behavioral costs are comparatively little given the benefit of ceasing the pain. However, if the organism has experienced little or no success with a certain active coping strategy in the past (for example, attacking the source of pain), and the situation does not allow for an alternative active strategy (e.g., flight), the most adaptive strategy is likely passive surrender (i.e., lower the costs) and pain endurance—the energy expenditure is minimized and the body gains time to heal (i.e., greatest anticipated benefit).

Similarly, if pain stems from an objectively inescapable source (e.g., visceral pain, inflammation), passive coping in the form of quiescence is likely to be adaptive. By reducing movement and allowing healing, quiescence might even help to avoid further damage. Beyond the concept of passivity as a default state of individuals who have not learned to control a certain stressor [put forward by Maier and Seligman (5)], we portray passive coping as an adaptive strategy when dealing with ongoing pain. Passive coping, including pain acceptance, encompasses engagement with the pain symptoms (6) and requires an active learning process. “Learned controllability” (5), for instance, can hinder pain acceptance—an individual who has learned to be generally in control of physical stressors may find it difficult to accept and endure pain. In contrast, once pain acceptance has been successfully acquired as a coping strategy, cognitive resources become available to focus on the surroundings. If incentives are present, it would be most adaptive to focus on their pursuit to offset the unpleasant sensation caused by ongoing, inescapable pain.

In this review, we present pain coping as an unconscious decision-making process during which accurately evaluated costs and benefits of behavioral choices lead to adaptive pain coping behavior. We also review the neurobiology underlying this behavior. Neural circuits involved in pain coping can trigger the dopaminergic system and thereby support vigilance toward rewarding stimuli while in pain. We argue that these mechanisms are of central importance to coping with ongoing, unavoidable pain. In the final part of the article we focus on alterations of the brain’s dopamine system as one of the neurobiological obstacles hindering adaptive coping in pain patients.

## Behavioral Findings for Active and Passive Pain Coping

### Active Escape from Acute Pain: From Reflexes to Complex Behaviors

Pain warns the organism of (potential) harm and calls for a quick coping action, e.g., the withdrawal of the affected body part from a harmful source or the escape from an unsafe environment. Ultimately, the organism will likely learn from the unpleasant experience and, in the future, adapt their behavioral response to the threat (e.g., an environment, a certain action, or a specific stimulus), which led to pain. Accordingly, Morrison and colleagues portrayed pain as an “action problem” (7) highlighting the necessity of immediate nocifensive behavior when facing potential injury. One of the simplest forms of nocifensive behavior is the withdrawal reflex. While reflexes provide a rapid initial action to prevent harm and to eliminate pain, more complex mechanisms allow for refined nocifensive behaviors (8). For example, early experiments in canines demonstrated that naïve dogs receiving escape-avoidance training show fear-like behavior in response to nociceptive electrical stimulation until the dog finally escapes to safety [e.g., jumping over a barrier to the safe compartment of a shuttle box (9)]. With every new exposure to the same aversive stimulation, the dogs showed the escape response faster and, thus, learned “mastery” (5). Similarly, human participants show faster reaction times during acute pain (10), suggesting a facilitation of the motor actions that would enable quick voluntary escape or avoidance responses. The neural underpinnings of this phenomenon are discussed in the Section “Neurobiological Correlates of Active and Passive Coping.”

### Passive Endurance of Escapable Pain: Adaptive and Maladaptive Behaviors

In certain situations, it is adaptive to endure pain even when it could be escaped and thus eliminated. For example, we might accept the pain of dental treatment because of the anticipated future benefit (e.g., increased dental hygiene, prevention of dental damage). The decision-making based on the comparison between costs and benefits/reward in the pain context has been formalized in the “Motivation-Decision Model” (11, 12). In this model, pain and reward are understood as interacting aversive and appetitive motivational systems. The individual (e.g., the patient at the dentist’s) has to prioritize one or the other, based on personal factors (How much do I value dental hygiene?), learning history (I survived the last treatment), and situational factors (Does the environment offer a way to escape the pain and still get the benefits, maybe in form of local anesthesia?).

While adaptive in certain situations, passive endurance of escapable pain can be maladaptive under different circumstances. About 50 years ago, the phenomenon of “learned helplessness” was first described (13). It refers to passive pain coping of animals in a situation where pain can be escaped and would be objectively more adaptive than passivity. However, the animals had been exposed to unavoidable nociceptive foot shocks in a different environment several hours before [e.g., Ref. (13–18)]. During this initial procedure of receiving inescapable shocks, the animals learn that shock termination is independent of their behavioral responses. Receiving shocks in a new environment, the expectation of not being in control is generalized, and the organism fails to learn appropriate escape behavior [reviewed in Maier and Watkins (19)]. The phenomenon of learned helplessness has been replicated experimentally in humans after exposure to inescapable noise [e.g., Ref. (20, 21)]. It was conceptualized that the person’s attributional style would determine whether the individual was likely to develop generalized helplessness after experiencing uncontrollability over a stressor (21, 22). According to this attributional theory of learned helplessness, people who attribute a lack of control over negative events to permanent causes (e.g., stress is always uncontrollable) and pervasive factors (e.g., most of my problems are unsolvable) are more vulnerable to generalize helplessness (5, 21–23).

Although it has not been directly tested, we posit that the phenomenon of learned helplessness is of crucial importance to the pain field. The treatment of pain often includes pharmacological approaches, but many patients still continue to experience substantial levels of pain (24) and often describe the treatment effects as unreliable and dissatisfactory (25). This leaves the patient with little or no control over their condition. As described above, learning that their own behavior has no consequence on the stressor (i.e., the pain) leads to learned helplessness in vulnerable individuals. Learned helplessness in humans has been associated with motivational and cognitive deficits, and emotional disturbances (22), which are indeed commonly described by pain patients (26–29).

The examples above highlight how similar behaviors in response to pain can be adaptive or maladaptive, depending on the accuracy of the individual’s appraisal of the relevant factors. Pain coping can, in fact, be described as a decision-making process based on the appraisal of costs and expected benefits (11, 12). In the example of the patient undergoing a dental procedure, costs and benefits are realistically anticipated, leading to the conclusion that benefits are greater than costs and therefore, tolerating pain is adaptive. In the instance of helplessness, however, costs are considered unrealistically high while—falsely—no or little benefit is being anticipated. This false appraisal of the cost/benefit ratio leads to unnecessary pain suffering, the perception of uncontrollability, and long-term emotional disturbances (22) and is thus considered maladaptive.

### Passive/Emotional Coping When Pain Is *Unavoidable*: Positive Pain Management, instead of Perseverance in Unsuccessful Attempts to Eliminate Pain

Even in many acute pain situations, escape or avoidance of the pain-provoking stimulus is not possible: an inflamed wound, toothache, or visceral pain are examples of acute pain situations that we are unable to escape from. Under these circumstances, it is often advantageous to engage in a passive/emotional coping style: the affected body part or the whole body is immobilized, and blood pressure and heart rate decrease (4, 30, 31), helping the body to rest and heal. One central concept within emotional coping strategies is *pain acceptance*. Though pain acceptance has sometimes been equated with “giving up,” it actually constitutes an active engagement with the pain. In contrast to helplessness, pain acceptance is based on an accurate appraisal of costs and benefits, with the realistic conclusion that the costs to fight (unavoidable) pain are too high given the low probability of benefits. It describes a readiness to accept the presence of pain and its inescapability, while no energy is being wasted to persevere in the attempt to change what cannot be changed (6, 24). Pain acceptance increases cognitive capacities and allows attending to rewarding events and stimuli in the environment while pain is ongoing. In the following paragraph, we discuss in more detail how reaching out for positive means describes an adaptive active coping style when dealing with unavoidable/inescapable pain.

### Reaching Out for Positive Means: Active Compensatory Coping When Pain Is Unavoidable

When inescapable/unavoidable pain is well managed *via* passive/emotional coping, cognitive capacities become available to focus on life-relevant and rewarding events. We classify the approach to screen the environment for incentives as an active pain coping attempt with the aim to reestablish the hedonic homeostasis, which is disturbed by the unpleasant experience of pain (1). Consistent with this, healthy individuals show increased motivation to obtain reward when in pain (32), presumably to compensate for the negative emotional state caused by pain and to reestablish the hedonic homeostasis. In our study, participants showed increased efforts (faster reaction times) to obtain high monetary reward when in pain compared to when they were pain-free. We concluded that vigilance for environmental cues with the potential to improve the hedonic state was increased by the ongoing pain stimulus. Comparable results have been found in rodents (33): acutely injured rats spent more time in near proximity to rewarding food pellets in the middle of an open field arena than control animals (33).

The fact that humans perform better when in pain is remarkable, given that pain demands attention and has been described as reducing the availability of cognitive resources (34–36). To resolve this apparent contradiction, it is important to consider whether the outcome of the task is of relevance to the participant’s state. In general, individuals are more sensitive to information that is pertinent to reach a certain goal; goal-irrelevant information, in contrast, is likely to be ignored [reviewed in Van Damme et al. (37)]. In analogy, while in pain, the sensitivity toward incentives associated with rewarding outcomes—as well as toward negative cues threatening to further worsen the homoeostatic imbalance—would be increased, because it assists in achieving the goal to reestablish hedonic homeostasis. Conversely, during goal-irrelevant tasks that do not impact the homeostatic balance while in pain, such as mental arithmetic, memory, or discrimination tasks, the performance is worse compared to a pain-free state, as shown in many experimental studies [e.g., Ref. (35, 38–40)]. Interestingly, experimental studies have shown that chronic pain patients (41–43) and healthy participants in acute pain (44) choose options associated with high immediate reward, while ignoring the fact that this choice is associated with higher risk and thus less advantageous in the long term. This fits the notion that ongoing or persistent pain makes individuals more sensitive to immediate rewards and less sensitive to long-term disadvantageous consequences. This phenomenon does not seem to be restricted to humans: rats with persistent inflammatory pain were shown to choose a lever associated with higher, but less frequent rewards over a safer option associated with smaller immediate rewards (45). We propose that individuals with ongoing pain focus on environmental cues that offer an opportunity to immediately improve their hedonic imbalance, i.e., high incentives, while higher-order processing of odds and potential risks is diminished. Furthermore, the experience of winning is itself analgesic (46, 47). Thus, reaching out for positive means—i.e., showing an increased effort to obtain reward—constitutes an active coping style with a twofold benefit when pain itself cannot be avoided or escaped: obtaining reward reestablishes or at least improves the hedonic homeostatic balance and reduces the perception of the pain. It is conceivable that such active compensatory coping might even occur when the rest of the body is in passive coping mode. That is, the affected body site or the whole body is kept at rest while the mind stays alert, screening the environment for incentives signaling the potential of immediate reward.

## Neurobiological Correlates of Active and Passive Coping

Studies on the neurobiology underlying pain coping have often used escapable and inescapable stimuli to investigate coping responses in animals. As expected, the former are typically associated with active escape responses, while the latter are related to passive strategies (4). However, as discussed above, organisms may react with passivity to stimuli that are objectively avoidable; and *vice versa* may use active coping strategies while healing from unavoidable pain is taking place (e.g., reaching out for positive means). In the following paragraphs, we discuss the neural substrates underlying the complex interacting influences of pain and individual circumstances on coping behavior.

### Coping in Response to Objectively Escapable Pain

Rodent studies demonstrate that generally, escapable acute pain, such as short-lasting superficial noxious stimulation of the skin and mainly mediated by A-delta fibers (e.g., pinches, pricking, and noxious heat stimulation), elicits active coping behaviors, accompanied by excitation of the sympathetic nervous system (31, 48, 49). One of the simplest forms of nocifensive behavior is the withdrawal reflex (7). At the dorsal horn of the spinal cord, the afferent nociceptive signal is conveyed directly or indirectly to spinal motor neurons to facilitate the withdrawal reflex. While reflexes provide a rapid initial action to prevent harm and to eliminate pain, more complex midbrain and cortex-regulated mechanisms allow for refined nocifensive behaviors. The dorsolateral periaqueductal gray (dlPAG) receiving mainly input from superficial A-delta fibers is key in orchestrating active coping responses (4, 30, 50). An important cortical site for integrating visual, auditory, and somatosensory information with movement toward or away from the harmful source is suggested to be the superior parietal cortex, as identified in a human fMRI experiment (51). To our knowledge, no evidence exists for direct connections between the dlPAG and the superior parietal cortex, but a human diffusion MRI study showed connectivity between these two regions (52). Therefore, dlPAG–parietal cortex connections might be the neural substrate of active nocifensive behavior in response to escapable pain in humans.

**Figure 2:**
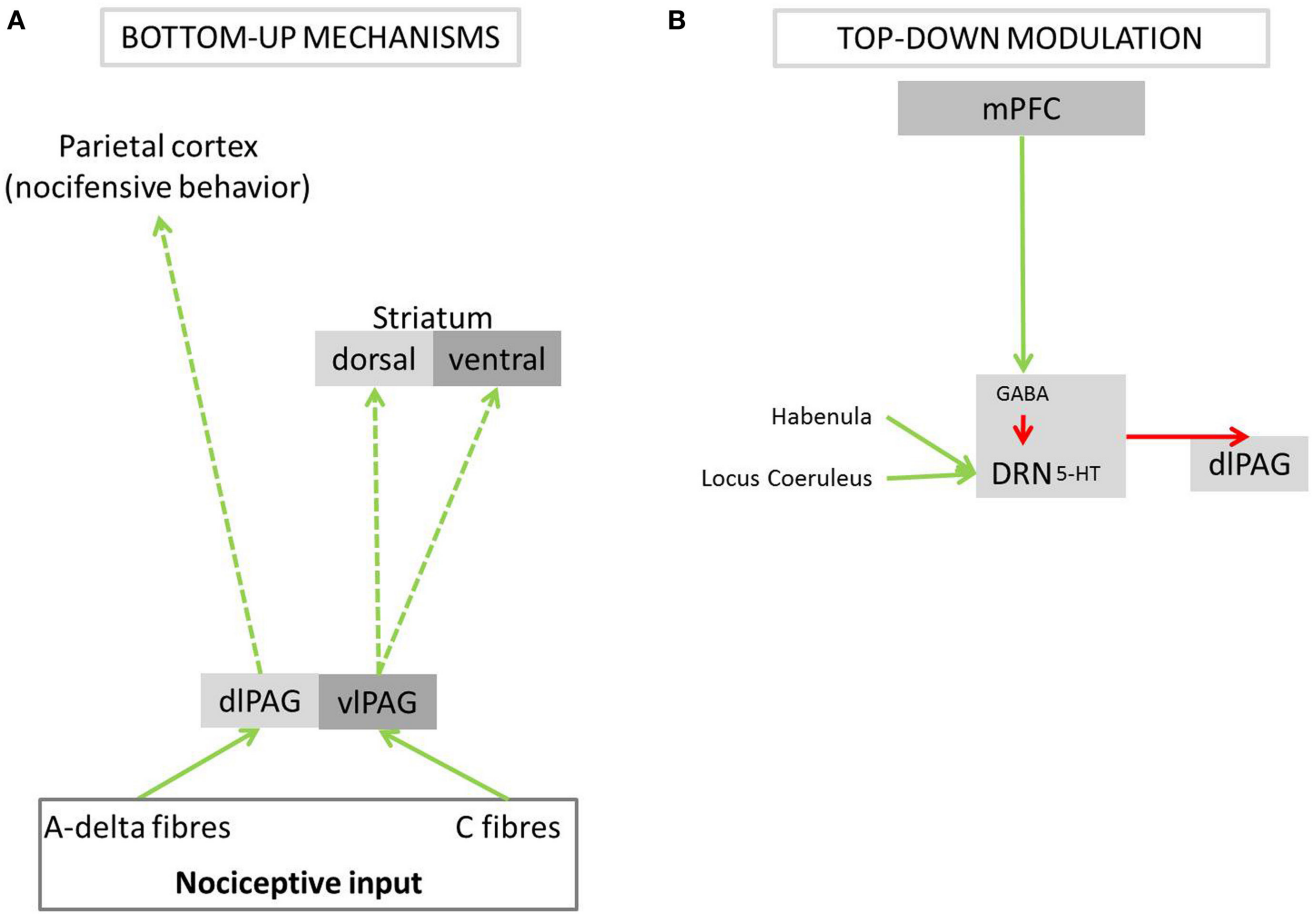
Bottom-up pathways of active and passive pain coping and its modulation by top-down mechanisms. The PAG is a central brain structure in pain coping, preparing the body for either fight/flight responses or passive endurance of the painful stimulus. **(A)** Different parts of the PAG receive nociceptive input from A-delta or C-fibers. **(B)** The PAG is modulated, *via* the DRN, by the mPFC, which signals subjectively perceived control over a painful event. PAG, periaqueductal gray; dlPAG, dorsolateral periaqueductal gray; vlPAG, ventrolateral periaqueductal gray; DRN, dorsal raphe nucleus; 5-HT, serotonin; GABA, gamma-aminobutyric acid; mPFC, medial prefrontal cortex; green arrows, excitatory activation; red arrows, inhibition; dashed arrows, anatomical connections *postulated* to underlie coping behavior in response to pain; solid arrows, anatomical connections *shown* to play a role in nociceptive processing or the modulation of behavior when expecting or experiencing pain.

Active pain coping triggered by A-delta input into the dlPAG is modulated by top-down mechanisms. The rodent literature shows that the medial prefrontal cortex (mPFC) evaluates the escapability of pain [reviewed in Maier and Seligman (5)]. If a stimulus is deemed escapable, the mPFC allows the dlPAG to exhibit an active coping response. Indirect projections have been shown to be important for active pain coping: inhibition of the dorsal raphe nucleus (DRN) by the mPFC disinhibits the dlPAG [reviewed in Maier and Seligman (5); Figure 2], thereby allowing fight or flight responses (4, 30, 50). In addition, direct anatomical connections between the mPFC and the dlPAG might also be involved in mediating active pain coping responses: the mPFC has been shown in rodents and macaque monkeys to project directly to the dlPAG (53, 54). In fact, such projections have recently been identified in mice as playing an important role for social defensive responses (55). In humans, the mPFC has been described to form and store schemata that integrate context, events, and appropriate action [reviewed in Euston et al. (56)]. The purpose of these schemata is to initiate the most suitable emotional and motor response to a given event, based on past experiences. Based on these lines of evidence, we speculate that also in humans, the dlPAG is disinhibited *via* the mPFC if the appraisal of pain results in the interpretation that it can be escaped, and consequently prepares the body for active coping.

In situations where the painful stimulus is not identified as escapable, the dlPAG and its functions are inhibited *via* neural networks identified in non-human studies (5). When pain is deemed inescapable, the mPFC does not inhibit the DRN, which is activated *via* excitatory nociceptive input from the habenula and locus coeruleus (57–59). Activity in the DRN suppresses activity in the dlPAG *via* serotonergic projections (60) and thereby triggers behavioral passivity. The serotonergic pathways from the DRN further activate the amygdala (61), which results in the emotional experience of fear.

The periaqueductal gray (PAG) also seems to code negative prediction errors, which has been described in humans using functional magnetic resonance imaging (62). Negative prediction errors in these circumstances describe the experience of a painful stimulus that was (falsely) evaluated as avoidable, i.e., the outcome was worse (more painful) than expected. This information is being fed back to the mPFC to update expectations for future pain events (62). This suggests that a similar pain event in the future will less likely be considered escapable; this, in turn will result in decreased dlPAG activation (*via* the top-down pathways described above) and therefore lead to decreased active escape behavior or even behavioral passivity.

### Coping in Response to Objectively Inescapable Pain

Objectively unavoidable acute pain, such as deep somatic or visceral pain, is conveyed by C-fiber input into the ventrolateral periaqueductal gray (vlPAG), which triggers passive coping behaviors and downregulation of the sympathetic nervous system in rodents (4, 30). Keay et al. (63), for instance, demonstrated that tonic nociceptive stimulation of the neck (deep muscle stimulation by 5% formalin solution or cutaneous stimulation by a neck clip) increased quiescence compared to control rats, which was associated with a selective increase of a marker of neural activity, c-Fos, in the vlPAG. As explained in the section above, serotonergic neurons of the DRN are activated simultaneously *via* the locus coeruleus and the habenula of the descending antinociceptive system (57–59). By activation of the raphe nucleus, serotonin is being released from axons in the spinal cord, which produces an analgesic effect by inhibiting incoming nociceptive signals [e.g., Ref. (64)]; the pain can thus be endured more easily. These mechanisms support passivity and quiescence of the body to allow healing and regaining of energy.

Of interest for this article are not only the afferent but also the efferent connections of the PAG, particularly to the dopaminergic structures ventral tegmental area (VTA) and substantia nigra identified by tracer studies in rodents (65–67). The PAG input to the nigrostriatal dopaminergic system *via* the substantia nigra pars compacta originates mainly in the ventral aspect of the PAG (65). Considering the central role of the nigrostriatal system in initiating and preparing motor responses demonstrated by lesion studies [e.g., Ref. (68, 69)], this system is a candidate substrate for increased motor performance in response to reward-associated stimuli while experiencing inescapable pain (see Reaching Out for Positive Means: Active Compensatory Coping When Pain Is Unavoidable). In contrast to the substantia nigra, the PAG input into the VTA does not seem to be restricted to one specific aspect of the PAG but originates from ventrolateral as well as dorsolateral columns (67). Within the VTA, these PAG efferents target dopaminergic neurons in the paranigral and the parabrachial subregions (67), providing a circuitry *via* which nociceptive input could trigger the mesolimbic dopaminergic system. In line with the anatomical circuitry, dopamine is released in the basal ganglia, including the striatum, in response to experimental pain in rats (70) and humans (71, 72). Dopamine release in response to acute pain has been described to serve the primary purpose to initiate pain-relevant behaviors helping the organism to either endure or avoid the pain depending on the situational circumstances [reviewed in Taylor et al. (73)]. Outside pain, dopamine plays a key role for reward processing (74–79): dopamine release is associated with enhanced awareness of stimuli signaling potential reward leading to increased motivation to obtain reward in animals and humans (74, 76, 80, 81). Thus, ventral striatal dopamine release in response to pain—presumably triggered by the PAG *via* the VTA—is well positioned to enhance the salience of incentives in the environment and thereby underlie the increased motivation to obtain reward observed in acute unavoidable pain. Considering that dorsolateral as well as ventrolateral columns of the PAG connect to the VTA, this might happen not only during coping with objectively inescapable pain but also during coping with escapable pain. In fact, dopamine release in the mesolimbic dopamine system of rats *predicts* successful avoidance of foot shocks (82), which can be interpreted as a positive expectation that the hedonic homeostasis will not be disturbed—in this instance, successful pain avoidance would have a similar reinforcement value as obtaining appetitive stimuli. In either instance—active or passive coping—dopamine release in the mesolimbic system would be an adaptive mechanism by which the brain tries to reestablish or maintain the affective homeostatic balance.

### Coping under Ambiguous Circumstances: Prediction and Control in Striatal–Cortical Circuitry

Above, we have relatively simplistically differentiated between escapable/avoidable and inescapable/unavoidable pain. In real life, pain stimuli lie on a spectrum of “avoidability.” For example, a certain price has to be paid to avoid pain or with a certain behavior pain can be decreased but not completely avoided. For any situation in which pain does not lie on either end of the spectrum, action selection and prioritization has to take place while accepting uncertainty about the costs/benefit ratio. In this section, we will discuss the neurobiology of these phenomena.

When pain is neither unambiguously escapable nor unambiguously inescapable, neural circuits in the central nervous system operate both to predict potentially noxious circumstances and to control behavior accordingly. These processes rely heavily on experience and learning and involve (voluntary) action selection. Depending on whether pain is acute (injury is experienced or anticipated), or whether it occurs in a post-injury context (in a healing phase), pain-related signaling will prompt different behavior and enlist different ensembles of neural circuits. Aversive responses during acute pain usually occur in the context of injury or threat of injury, referred to here as “peri-injury” pain processing. Generally, a “go” decision—escape or avoidance—is optimal in peri-injury pain processing, because the benefit of pain elimination seems to outweigh the behavioral cost. In contrast, “post-injury” pain processing occurs after tissue has been damaged, and behavior that promotes healing is prioritized, including quiescence and inhibition of normal behavioral activity (any attempt to eliminate the pain, i.e., behavioral cost would be greater than the benefit). Generally, a “stay” decision—rest and recuperate—is therefore optimal in post-injury pain processing.

Neural mechanisms of prediction and control in peri-injury processing involve the striatal dopamine circuits discussed above, as well as cortical circuits with which they interact. This involves instrumental reinforcement learning about actions and outcomes. For example, when an action carries both costs and benefits, responses in ventral ACC and ventral striatum are attenuated when the predicted costs outweigh the benefits—this attenuation increases with an increase of the negative relation of costs over benefits (83). The greater the amount of additional information needed to select an appropriate action, the greater the demand for higher-order levels of control *via* cortical networks. In a hierarchical “cascade,” various nested levels are subsumed, ranging from immediate context, memory, and any relevant contingencies or rule-based information (84). This results in more flexible and discretionary forms of control, going beyond reflex action or the more binary on–off control of inhibitory feedback loops. Subregions of the ACC probably work together to integrate stimulus content and current task demands to produce appropriate and timely responses (85, 86). These mechanisms are likely to be critical for active coping in the face of pain.

There is evidence that simpler levels of the control hierarchy in cingulate cortex are located more caudally (for example, in dorsal posterior cingulate cortex), and increasingly more nested and complex levels in the more rostral direction [for example, the rostral, perigenual portions of the dorsal ACC (7, 87)]. Recent human neuroimaging evidence indicates that voluntary motor-related processing can account for MCC activation during pain, particularly in the caudal cingulate motor zone (CCZ) (10). Rostral ACC regions are particularly densely interconnected with dorsomedial and dorsolateral prefrontal networks that are also implicated in executive processing and action selection. These areas may contribute to a ranking of choices in both current and prospective temporal windows (88).

ACC and insular cortices are interconnected and highly coactive during pain, as well as in a range of other contexts (89). In situations involving acute pain, predictive dynamics between ACC and anterior insula (AI) probably reflect processing related to assigning priority to a stimulus and selecting an action in voluntary responses to pain (7). The organization of pain processing shows evidence of a caudorostral gradient in the insula, in terms of connectivity and roles in distinct cortical networks (90, 91), reaching a high degree of integration in AI (92). Importantly, this integration probably occurs in a predictive manner, with AI activity predicting whether a subject will classify a stimulus as painful even before the stimulus occurs (93).

These cortical networks may also contribute to coping strategies. For example, the vlPAG is functionally connected with rostral ACC even during rest, when the brain is not receiving nociceptive input nor engaged by a task (94). In addition to the striatal functions described above, signaling in the striatum when anticipating pain may signal the expected value of a painful stimulus to the PAG, which communicates this signal to a wider cortical circuit that includes control-related regions of dACC (62). This is consistent with the PAG instigating dopamine release in the striatum and “energizing” active responses to ongoing pain. Dopamine-dependent circuits crucially support predictions about reward (95) as well as playing a central role in the implementation of such learning in the instrumental control of action.

The interesting case with respect to coping is when an individual makes active, peri-injury-like decisions under chronic or post-injury circumstances. What determines the shift from a passive to active coping mode in chronic pain? Individual differences in PAG–striatum–cortical network dynamics may play a role. For acute, unavoidable painful stimulation, healthy individuals differ in their learning biases (96): some individuals displayed a “negative” learning pattern in which pain drives aversive learning, while others displayed a “positive” pattern in which learning is driven more by success in avoiding pain. These individual differences were associated with differential responses in the striatum and were predicted by striatal gray matter density (96). The complex striatocortical architecture of control and prediction neurocircuitry in pain behavior reflects the fact that broad behavioral strategies like “go” or “stay” can be generally effective under certain sets of circumstances but may not necessarily prove beneficial with respect to pain outcomes in any given situation, for example, as when subjective pain outcomes in chronic pain would improve by shifting to an active strategy.

## Coping and Chronic Pain

Thus far, we have presented fundamental principles of pain coping and its underlying neurobiology. In the last section of this article, we discuss the relevance of these concepts and neural circuitries for chronic pain in more detail.

Per definition, chronic pain persists or recurs over a period longer than 3 months (97). This means that even for patients who have some control over their pain and can reduce or even avoid pain under certain circumstances, pain episodes are frequent and/or long enough to qualify as chronic pain. Nevertheless, it would be too simple to view chronic pain merely as being unavoidable. How avoidable chronic pain episodes are perceived as encompasses a wide spectrum. Some types of chronic pain lie at the extreme end and are practically unavoidable, such as non-evoked neuropathic pain. Other types, such as movement-related pain in osteoarthritis, might be largely avoidable, as long as the individual adjusts his or her behavior accordingly. These two examples illustrate the two major goals of coping with chronic pain: pain reduction on the one hand and on the other hand, the continued pursuit of valued activities and life goals (2), despite pain.

As the example of movement-related pain, the goals of pain reduction and the continued pursuit of valued activities may be incompatible with each other and may be associated with conflicting motivations. Therefore, the need for valuation, goal setting, goal adjustment, and goal pursuit strategies becomes extremely important in the management of chronic pain. These are all processes necessitating active engagement of the patient. Indeed, active coping strategies in chronic pain patients with a focus on the pursuit of life-goals are associated with improved health and treatment outcomes (98, 99). Active coping in the context of chronic pain refers to accepting responsibility for dealing with one’s pain and to be actively engaged in developing strategies for pain reduction and the pursuit of other life goals. The best care strategies for chronic pain require the patient to embrace lifestyle adaptations and self-management (100). Of course, active coping is not to be confounded with “activity no matter what.” As discussed above, it is central to adaptive pain coping that costs and benefits are realistically appraised, while benefits should outweigh costs. In order to keep the costs within an appropriate range given the benefits, self-management encompasses activity pacing, in addition to behavioral persistence (101). Although emotional coping strategies are grouped under avoidant, and therefore passive, coping (102), we suggest that they are necessary to allow for active coping when unavoidable pain is present. Above, we introduced the concept of pain acceptance (i.e., a readiness to accept the presence of unavoidable pain, while not wasting energy on unsuccessful attempts to eliminate it) as an example of passive/emotional coping. This concept focuses on increased functionality despite having pain and enables the patient to focus on rewarding events in their environment. In fact, among chronic pain patients, the self-efficacious ones could be demonstrated to show less depressive symptoms and their pain interfered less with daily-life activities (103–106). We conclude that emotional coping is a central initial process, necessary to allow for adaptive active coping with chronic pain, for instance, by means of focusing on pleasurable events and increased functionality.

### Neurobiological Obstacles for Active Coping With Chronic Pain

If a combination of pain acceptance and active (goal-pursuing) coping strategies are most adaptive for dealing with chronic pain, why do many patients persevere in their passivity, while avoiding the next important step of an active approach (101)? The answer encompasses different domains, ranging from psychological to neurobiological to societal phenomena. Psychological obstacles comprise, but are not limited to, avoidant behavior, including learned avoidance (a certain behavior is punished by experiencing pain), misinformed avoidance (“bending over is bad”), and affective avoidance (fear of pain) (107), learned helplessness (underestimating the benefit/cost ratio based on the individual learning history), and misguided reinforcement (e.g., pain behaviors are rewarded). Excellent reviews on psychological aspects of obstacles to coping with chronic pain have been published (107, 108); we attempt here to integrate this with neurobiological findings emerging in the chronic pain literature.

Several brain functions are required for active coping, such as an evaluation of the situation integrating past experiences (5), action selection (56), control of affect (5, 56), and goal adjustment. Not all these functions have been experimentally assessed in chronic pain patients; however, patients have been described to present with cognitive difficulties, including impaired decision-making (41–43). Further, it has become clear in recent years that dorsomedial and dorsolateral parts of the prefrontal cortex—each an integral component within striatal–cortical control networks—display structural and functional alterations in chronic pain patients [reviewed in Bushnell et al. (109) and Smallwood et al. (110)]. Thus, cognitive functions important for active coping might be impaired in chronic pain patients due to brain alterations, which would be an important obstacle to adaptive coping. In addition, the mPFC is important for classifying a stimulus as being avoidable or controllable, as described above. A dysfunctional mPFC might be less capable of identifying when pain is controllable, leading *via* the DRN to an inhibition of the dlPAG. This in turn would impede active coping. Several lines of evidence indicate that structural brain changes are at least partly a consequence of pain or prolonged nociceptive input (111) and this could initiate a viscous circle or even a downward spiral of pain and unsuccessful coping.

A number of other neurobiological factors hinder successful active coping in chronic pain. As discussed above in Section “Neurobiological Correlates of Active and Passive Coping,” active versus passive coping responses are influenced by bottom-up afferent input with C-fibers preferentially triggering passivity. Of course, chronic pain constitutes a heterogeneous group of different conditions, comprising among others neuropathic and inflammatory pains. Despite this heterogeneity, C-fibers, in particular from deep tissues and viscera, likely provide important afferent input in many chronic conditions. For example, central sensitization and wind-up, considered to be two important mechanisms operating in neuropathic pain, depend on C-fiber input into the spinal cord (112, 113). Under conditions of inflammation, “silent” nociceptors that, in healthy tissue, are mechanically insensitive and not activated even by strong stimuli, become excitable to pressure, changes in temperature, and tissue acidosis, which contributes to the generation and maintenance of hyperalgesia (114). In the joint, approximately one-third of sensory C-fibers and a small percentage of A-delta fibers are estimated to be mechano-insensitive silent nociceptors (114). Thus, the neurobiological coping response triggered by bottom-up input ought to be passivity in many chronic pain conditions, even when the pain is not accompanied by significant emotional distress. To perform active coping successfully, chronic pain patients thus have to overcome this neurobiology that defaults the individual to passivity. In addition to C-fiber input promoting passivity, we and others have postulated that an “inactivity response” operates in some chronic pain patients that resembles the sickness response observed with systemic inflammation (115, 116). Such a response consisting of widespread pain, hypersensitivity to somatosensory as well as auditory or olfactory stimuli, stiffness, fatigue, lethargy, and depressed mood can be understood as an integrated program designed to force the organism to rest (116). This program would be beneficial and promote healing and recovery in situations of acute inflammation, infection, or certain types of visceral pain. However, activation of the inactivity responses by, e.g., psychological stressors or regional pain problems would be maladaptive, because quiescence neither leads to the elimination of the stressor in these situations nor does it improve the hedonic state or functionality. In many instances, passivity even aggravates the problem through deconditioning and withdrawal. Intriguingly, the symptom constellation of the inactivity response—widespread pain particularly in deeper tissues, hypersensitivity to somatosensory as well as auditory or olfactory stimuli, stiffness, fatigue, lethargy, and depressed mood—corresponds to the clinical picture of fibromyalgia and related “primary” pain conditions (97). The role nociceptive input from the periphery plays in primary pain conditions remains debated (97) and the potential neurobiology of the postulated inactivity response is currently unknown. Extrapolating from the classical sickness response, however, suggests that neuroinflammation might be a candidate mechanism (115). Activation of glia cells, an important constituent of neuroinflammation, has indeed been observed in the brains of patients with chronic low back pain (117).

Activated glia and an associated pro-inflammatory cytokine response in the CNS are known to have substantial effects on various neurotransmitter systems (118). Regarding the monoamine transmitters, which include serotonin, norepinephrine, and dopamine, cytokines impact on synthesis, re-uptake, and release, typically with the net effect of decreasing monoamine availability in the brain [reviewed in Miller et al. (118)]. Dopaminergic neurons in the substantia nigra have been shown to be particularly sensitive to inflammation (119), which is related to the high density of microglia in this region. This is interesting because it has become clear in recent years that chronic pain is associated with changes in the brain’s dopamine systems [reviewed in Taylor et al. (73)]. Human imaging studies have found lowered responsiveness within the mesolimbic as well as the nigrostriatal dopamine system in response to salient stimuli in chronic pain patients (120–123). Chronic pain patients have lower D2 receptor binding (121, 123–125) and presynaptic dopamine activity (126, 127) in the striatum at rest and following an acute pain stimulus. In animal studies, chronic pain results in decreased neuronal activity, as assessed by c-Fos staining, in the VTA (128), and decreased overall dopamine levels and striatal D2 receptors (129–132). Given the importance of dopamine for motivated behavior (133), it is not surprising that chronic pain animals have been observed to show reduced effort to obtain a food reward, which was linked to depressed activity in the indirect pathway of the ventral striatum (134). Other functions associated with dopamine have similarly been found to be impaired in chronic pain animals, for example, exploratory behavior (135) and self-administration of rewarding opioids (136); although this has not yet been directly linked to dopamine dysfunction. Considering the importance of the dopaminergic system for goal-directed behavior and reward processes in general, impairments in this system are likely to impact successful coping. Although the circuitries for passive pain coping are strongly engaged in patients while experiencing uncontrollable pain, the associated triggering of the reward system *via* vlPAG and ventral striatum does not lead to the beneficial effects described for healthy individuals (i.e., improving hedonic tone, reducing pain, and promoting health), likely because of the various changes in the brain’s dopamine systems associated with chronic pain (73).

To summarize, active strategies in addition to pain acceptance are key for successful coping with chronic pain. But as discussed in this section, there are several neurobiological, in addition to psychological, obstacles to active coping, making the implementation of the most beneficial behaviors more difficult. Neurobiological obstacles include the importance of C-fiber input in chronic pain because it is wired to elicit passivity without leading to the associated benefits of increased vigilance toward rewarding stimuli due to alterations of the brain’s dopamine system in chronic pain patients. Supraspinal circuits important for action selection, goal pursuit, and (perceived) control are likely impaired in chronic pain patients, contributing to a viscous circle of pain and unsuccessful coping. And finally, an inactivity response might be inappropriately activated in some chronic pain patients, reflected in complex symptom constellations consisting of widespread pain, hypersensitivity, fatigue, and depressed mood. For successful coping, these difficulties have to be overcome. An understanding of the neurobiological obstacles might be helpful for the health-care provider to understand and accept when patients display passivity. In addition, patients might benefit from learning about the neurobiological basis of coping and their difficulties to adopt active strategies, similar to the effectiveness of pain neuroscience education in reducing pain, improving function, and lowering disability (137).

## Author Contributions

All authors listed have made substantial, direct, and intellectual contribution to the work and approved it for publication.

## Conflict of Interest Statement

The authors declare that the research was conducted in the absence of any commercial or financial relationships that could be construed as a potential conflict of interest.
